# Structural characterization of TIR-domain signalosomes through a combination of structural biology approaches

**DOI:** 10.1107/S2052252524007693

**Published:** 2024-08-27

**Authors:** Akansha Bhatt, Biswa P. Mishra, Weixi Gu, Mitchell Sorbello, Hongyi Xu, Thomas Ve, Bostjan Kobe

**Affiliations:** a Institute for Glycomics Griffith University Southport QLD4222 Australia; b School of Pharmacy and Medical Sciences Griffith University Southport QLD4222 Australia; c School of Chemistry and Molecular Biosciences University of Queensland Brisbane QLD4072 Australia; d Institute for Molecular Bioscience The University of Queensland Brisbane QLD4072 Australia; e Australian Infectious Diseases Research Centre The University of Queensland Brisbane QLD4072 Australia; f Department of Materials and Environmental Chemistry Stockholm University Stockholm Sweden; University of California, Los Angeles, USA

**Keywords:** Toll/interleukin-1 receptor, signalosomes, innate immunity, micro-electron diffraction, helical reconstruction, serial femtosecond crystallography

## Abstract

The TIR (Toll/interleukin-1 receptor) domain represents a vital structural element shared by proteins with roles in immunity signalling pathways across phyla (from humans and plants to bacteria). Decades of research have finally led to identifying the key features of the molecular basis of signalling by these domains, including the formation of open-ended (filamentous) assemblies (responsible for the signalling by cooperative assembly formation mechanism, SCAF) and enzymatic activities involving the cleavage of nucleotides. We present a historical perspective of the research that led to this understanding, highlighting the roles that different structural methods played in this process: X-ray crystallography (including serial crystallography), microED (micro-crystal electron diffraction), NMR (nuclear magnetic resonance) spectroscopy and cryo-EM (cryogenic electron microscopy) involving helical reconstruction and single-particle analysis. This perspective emphasizes the complementarity of different structural approaches.

## Abstract 2

The TIR (Toll/interleukin-1 receptor) domains are found in proteins with roles in the immune systems of humans, plants and bacteria. A combination of structural methods ranging from X-ray and electron crystallography to cryogenic electron microscopy and nuclear magnetic resonance spectroscopy has been required to understand how these domains contribute to signalling, highlighting the complementarity of different structural approaches.

## 1. Introduction

The Toll/interleukin-1 receptor (TIR) domains were first described in the early 1990s as regions displaying sequence similarity between the mammalian interleukin-1 receptor (IL-1R) and the Drosophila Toll protein (Gay & Keith, 1991 ▸; Schneider *et al.*, 1991 ▸). IL-1Rs and Toll have roles in development and innate immune signalling (Ip *et al.*, 1993 ▸; Lemaitre *et al.*, 1996 ▸; Rutschmann *et al.*, 2000 ▸). Subsequently, TIR domains have been found in proteins with immune system roles across the domains of life (Essuman *et al.*, 2022 ▸; Ve *et al.*, 2015 ▸).

In animals, in addition to the IL-1R family, TIR domains are found in Toll-like receptors (TLRs; 10 family members in humans: TLR1-10) and cytosolic adaptor proteins. TLRs are pattern-recognition receptors (PRRs) that provide protection against microbial infection and endogenous danger by interacting with conserved pathogen-associated and danger-associated molecular patterns (PAMPs and DAMPs) (Fitzgerald & Kagan, 2020 ▸). Upon PAMP or DAMP recognition, TLRs recruit the downstream adaptor proteins MyD88 (myeloid differentiation primary response gene 88), MAL (MyD88 adaptor-like/TIRAP, TIR domain-containing adaptor protein), TRIF (TIR domain-containing adaptor protein inducing IFNβ/TICAM-1, TIR-containing adaptor molecule-1) and TRAM (TRIF-related adaptor molecule/TICAM-2) via TIR:TIR domain interactions (Luo *et al.*, 2019 ▸). Recruitment of these adaptors enables further downstream signalling that ultimately leads to pro-inflammatory cytokine and interferon production. Another TIR domain-containing adaptor protein, SARM1 (sterile alpha and TIR motif-containing 1), was first described as a negative regulator of TLR signalling, but has additionally emerged, over the past decade, as a key executioner of axon degeneration upon injury and disease (Coleman & Höke, 2020 ▸; DiAntonio *et al.*, 2021 ▸; McGuinness *et al.*, 2023 ▸).

In plants, TIR domains are found in a class of intracellular nucleotide binding, leucine-rich repeat receptors (NLRs), which trigger a cell-death response commonly referred to as effector-triggered immunity (ETI), upon direct or indirect recognition of pathogen effector proteins (Maruta *et al.*, 2022 ▸, Chai *et al.*, 2023 ▸). TIR domains are also widely found in bacteria and archaea and feature in diverse anti-phage defence systems (Hogrel *et al.*, 2022 ▸; Koopal *et al.*, 2022 ▸; Morehouse *et al.*, 2022 ▸; Tal *et al.*, 2021 ▸; Johnson *et al.*, 2022 ▸; Doron *et al.*, 2018 ▸), as well as in virulence factors that interfere with TLR signalling in animals and ETI in plants (Cirl *et al.*, 2008 ▸; Eastman *et al.*, 2022 ▸; Li *et al.*, 2023 ▸).

TIR domains have two key molecular functions. In animal innate immune responses involving TLRs or IL-1Rs, TIR domains facilitate the assembly of signalling complexes. By contrast, the TIR domains of SARM1, plant NLRs and bacterial anti-phage defence systems feature self-association dependent nucleotide (mainly NAD^+^, nicotinamide adenine dinucleotide – the oxidized form) hydro­lase activity. SARM1 and TIR domains of some anti-phage defence systems, including the CBASS (cyclic oligonucleotide based anti-phage signalling), the PYCSAR (pyrimidine cyclase system for anti-phage resistance) and the SPARTA (short prokaryotic argonaute TIR-APAZ) defence systems, rapidly deplete axons and cells of NAD^+^, resulting in axon degeneration and death of phage-infected bacterial cells, respectively (Essuman *et al.*, 2017 ▸; Hogrel *et al.*, 2022 ▸; Koopal *et al.*, 2022 ▸; Morehouse *et al.*, 2020 ▸; Tal *et al.*, 2021 ▸). TIR domains of plant NLRs and the anti-phage defence system Thoeris, on the other hand, use NAD^+^ as a substrate to produce immune signalling molecules (Huang *et al.*, 2022 ▸; Jia *et al.*, 2022 ▸; Ofir *et al.*, 2021 ▸; Li *et al.*, 2023 ▸; Manik *et al.*, 2022 ▸). Both processes may operate jointly in some cases (Li *et al.*, 2022 ▸).

**Figure 1:**
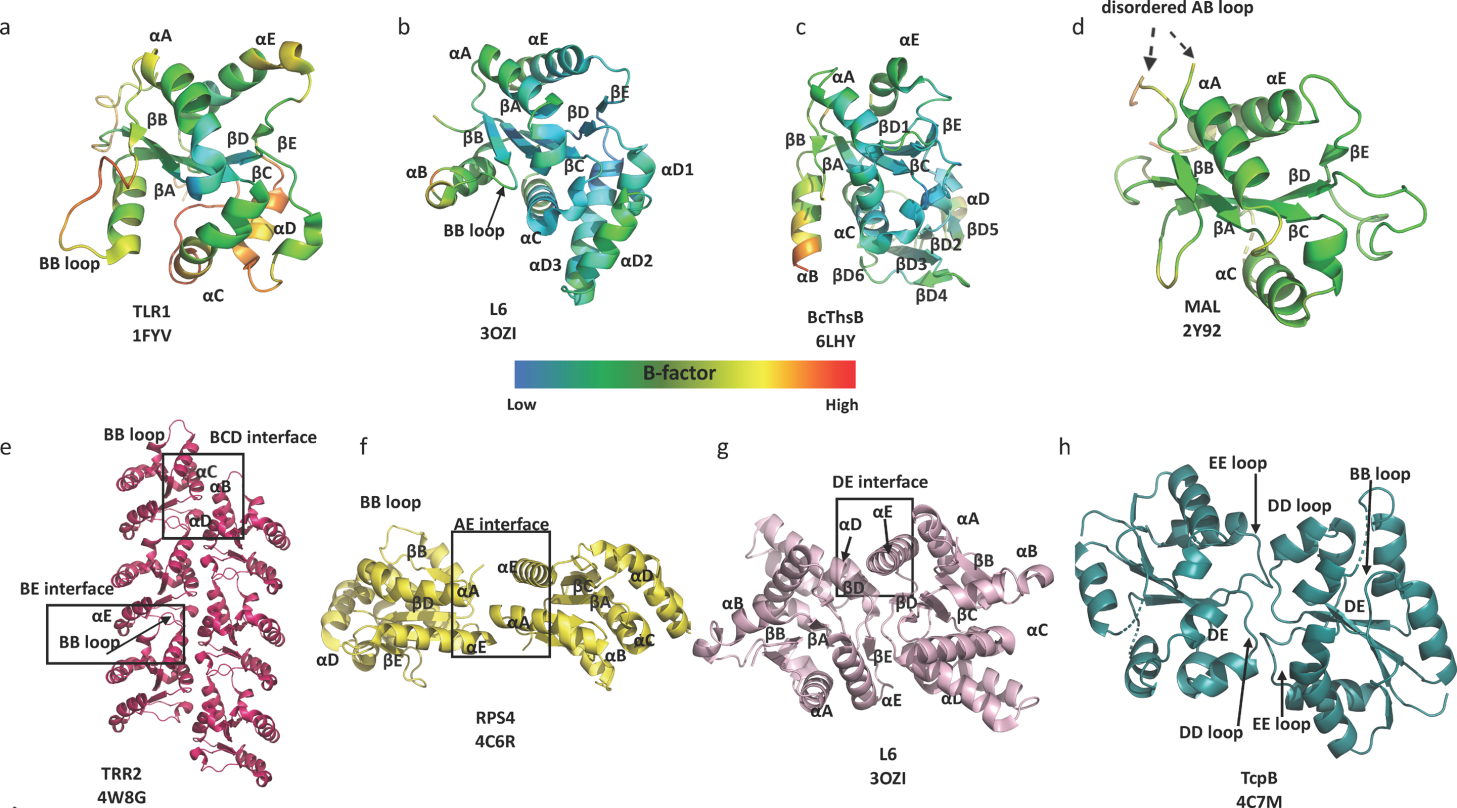
Representative TIR domain crystal structures. (*a*)–(*d*) Structures of TIR domains (coloured depending on their *B* factors) from (*a*) TLR1, (*b*) L6, (*c*) BcThsB and (*d*) MAL are represented as ribbons. (*e*)–(*h*) Crystal contact based TIR-domain assemblies observed in the structures of (*e*) TRR2 (magenta), (*f*) RPS4 (yellow), (*g*) L6 (pink) and (*h*) TcpB (teal). The BE interface involves residues from the BB loop of one TIR domain subunit and residues from the βD strand, βE strand and αE helix of the next subunit. The BCD interface involves residues from the αC helix and either the αB/BB-loops or the αD region, or both. The AE interface involves residues in the EE loop, αA helix and αE helix of both subunits, whereas the DE interface predominantly involves residues from the αD and αE helices. The DD/EE loop interface involves the DD and EE loops.

The first TIR-domain crystal structures, the structures of the TLR1 and TLR2 TIR domains, were reported by Liang Tong’s group in 2000 (Xu *et al.*, 2000 ▸) [Fig. 1 ▸(*a*)]. Since then, a wealth of information on TIR domain structure has been published, using a variety of structural biology methods including X-ray crystallography, nuclear magnetic resonance (NMR) spectroscopy, cryo-electron microscopy (cryo-EM) and microcrystal electron diffraction (microED), which have led to a detailed understanding of how TIR domains self-associate to form both signalling complexes and active enzymes capable of cleaving nucleotides; how TIR domain enzyme activity can be regulated; and the chemical structures of immune signalling molecules that they produce. Here, we review how structural biology has led to the understanding of the TIR domain function, from a mostly historical perspective (which to a large extent also corelates with the methodologies employed and the protein samples studied – isolated domains, larger complexes and filaments), and highlight how different approaches have contributed to this understanding. The overview highlights the complementarity of structural approaches and how their integration was required to obtain a holistic picture of the function of TIR domains in immunity and cell-death pathways.

## 2. Studies of isolated TIR domains by X-raycrystallography

The vast majority of detailed 3D structural information on TIR domains has come from single-crystal X-ray diffraction studies. In total, 74 TIR domain structures solved by X-ray crystallography have been deposited in the PDB (Protein Data Bank; Table S1 of the supporting information). These include the TIR domains from: TLR1, 2, 6 and 10; several IL-1Rs; the TLR signalling adaptors MAL, MyD88 and SARM1; several plant NLRs; and bacterial proteins with functions in virulence and anti-phage defence. To facilitate crystallization, crystallographic studies focused almost exclusively on TIR domains only. The structures of TLR1 and TLR2 TIR domains (Xu *et al.*, 2000 ▸) revealed that TIR domains have a flavodoxin-like fold with a central parallel β-sheet core surrounded by α-helices and loop regions, with α-helices and β-strands alternating throughout the protein sequence [Figs. 1 ▸(*a*)–1 ▸(*d*)]. The elements of the secondary structure are therefore usually referred to sequentially; for example, the BB loop connects strand βB with helix αB. The highly conserved and functionally important BB-loop region extends away from the rest of the TIR domain, forming a protrusion on the surface of the structure.

Although all TIR domains share the flavodoxin-like fold, significant differences are observed in some of the loop and α-helical regions. For example, most plant TIR domains have an extended αD helical region (Bernoux *et al.*, 2011 ▸) [Fig. 1 ▸(*b*)], whereas BcThsB, a TIR domain-containing protein from the Thoeris anti-phage system in *Bacillus cereus*, has an additional β-sheet domain inserted into the DE loop region (Ka *et al.*, 2020 ▸) [Fig. 1 ▸(*c*)]. Intriguingly, the crystal structure of the TIR domain from the TLR adaptor MAL is significantly different from TIR domains from MyD88 and the TLRs. The structure lacks the αB helix and features a long AB loop situated between helix αA and β-strand βB, which includes the conserved and functionally important BB-loop proline-containing motif (Lin *et al.*, 2012 ▸; Valkov *et al.*, 2011 ▸; Snyder *et al.*, 2014 ▸; Woo *et al.*, 2012 ▸) [Fig. 1 ▸(*d*)]. Moreover, the structure is stabilized by two intramolecular di­sulfide bonds involving residues C89–C134 and C142–C172, which is unusual for a cytoplasmic protein.

### 2.1. TIR domain self-association: insights from crystal contact analyses

TIR domains signal through self-association and homotypic association with other TIR domains. However, most TIR domains expressed on their own behave as monomers during purification. As crystal contacts can reflect biological interactions (Kobe *et al.*, 2008 ▸), many of the crystal structures were analysed for possible TIR-domain self-association modes; in combination with site-directed mutagenesis and computational modelling, several models for assembly of TIR domain signalling complexes were proposed [reviewed by Nimma *et al.* (2017 ▸)]. Although these models were all different from each other, some common trends in TIR-domain interaction modes emerged for mammalian TIR domains: the BCD interface, which involves residues from the αC helix and either the αB/BB-loops or the αD region, or both; and the BE interface, which involves the BB-loop of one TIR domain subunit and the surface encompassing the βE/EE loop/αE region of a second subunit. In one of the structures of Toll-related receptor TRR-2 from the lower metazoan *Hydra magnipapillata* (PDB entry 4w8g; Scheidig & Weisse, to be published), these interfaces were both observed in the crystals, leading to the formation of linear two-stranded arrays of TIR domains [Fig. 1 ▸(*e*)], with an analogous architecture to higher-order assembly structures of TIR domains determined subsequently using cryo-EM and microED (described in detail below).

Plant TIR domains with cell-death activities similarly self-associate to exert their function. Analyses of crystal contacts led to the identification of two functionally important self-association interfaces: the AE interface, which involves residues from the αA and αE helices [Fig. 1 ▸(*f*)]; and the DE interface, which involves residues from the αD_1/3_, βE and αE regions [Fig. 1 ▸(*g*)] (Bernoux *et al.*, 2011 ▸; Williams *et al.*, 2014 ▸, 2016 ▸; Zhang *et al.*, 2017 ▸).

In bacterial TIR proteins, a common self-association interface involving the DD and EE loops has been observed in the crystal structures of the TIR domains from *Paracoccus denitrificans*, PdTLP; *Brucella melitensis*, TcpB; and *Acinetobacter baumanii*, AbTir (Alaidarous *et al.*, 2014 ▸; Chan *et al.*, 2009 ▸; Manik *et al.*, 2022 ▸; Snyder *et al.*, 2014 ▸; Kaplan-Türköz *et al.*, 2013 ▸) [Fig. 1 ▸(*h*)]. TcpB is involved in virulence (Cirl *et al.*, 2008 ▸) and has been proposed to dampen mammalian innate immune responses by disrupting TIR:TIR domain interactions required for TLR signalling (Alaidarous *et al.*, 2014 ▸; Snyder *et al.*, 2014 ▸). The DD/EE loop interface is not conserved in crystal structures of TIR domains involved in anti-phage defence (Ka *et al.*, 2020 ▸; Morehouse *et al.*, 2020 ▸), and has not been observed in mammalian and plant TIR domains.

## 3. Studies of isolated TIR domains by NMR spectroscopy

**Figure 2:**
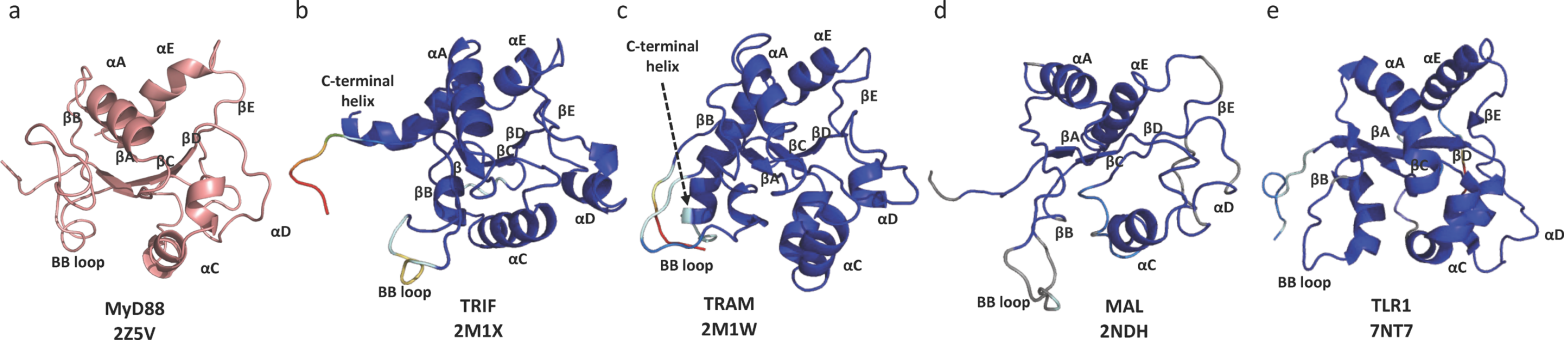
Representative TIR domain NMR structures. The structures of TIR domains from (*a*) MyD88, (*b*) TRIF, (*c*) TRAM, (*d*) MAL and (*e*) TLR1 are shown in cartoon representation and coloured by random coil index (RCI) (blue for most compacted, red for most flexible/disordered). The structure of the TIR domain from MyD88 is shown in pink due to the unavailability of RCI values from the PDB.

Because the size of TIR domains is amenable to NMR spectroscopy, this complementary technique has been employed in a number of cases for structure determination of TIR domains, some of which presumably could not be crystallized: the TLR signalling adaptors MyD88, MAL, TRIF, TRAM and the receptor TLR1 (Ohnishi *et al.*, 2009 ▸; Enokizono *et al.*, 2013 ▸; Hughes *et al.*, 2017 ▸; Lushpa *et al.*, 2021 ▸) (Table S1; Fig. 2 ▸). NMR structures of MyD88 TIR domain (PDB entries 2js7; Rossi *et al.*, to be published; 2z5v; Ohnishi *et al.*, 2009 ▸) are very similar to the reported crystal structure [Fig. 2 ▸(*a*)]. Ohnishi *et al.* (2009 ▸) attempted to map the interaction of the ^15^N-labelled MyD88 TIR domain with its interacting partner MAL, using NMR titrations, but the ^15^N signal uniformly decreased upon titration of MAL TIR domain, which was most likely due to increased apparent molecular weight upon complexation. Although a MyD88:MAL interface could not be identified in these studies, the formation of higher molecular weight complexes is consistent with more recent observations (see below).

Purified TIR domains of TRIF and TRAM aggregated at a concentration of 200 µ*M*, making NMR experiments difficult. To make these proteins more soluble, mutations were introduced in the BB-loop (P116H in TRIF and C117H in TRAM; these mutations block TLR signalling) (Enokizono *et al.*, 2013 ▸). Both structures adopt the characteristic TIR domain fold with an additional C-terminal helix [Figs. 2 ▸(*b*)–2 ▸(*c*)]. In both structures, the conformation of the BB-loop was not well defined due to broadening of the NMR signals, resulting in insufficient NOE distance restraints.

Hughes *et al.* (2017 ▸) reported the NMR structure of the MAL TIR domain [Fig. 2 ▸(*d*)]. As MAL is also prone to self-association and forms filaments at 25°C under physiological buffer conditions (described in detail below), the authors introduced a mutation (C116A) that limited TIR domain oligomerization. NMR data were collected at 18°C in a buffer with a pH of 8.6 and salt concentration of 200 m*M* NaCl. These conditions are considered extreme for NMR and are typically not suitable for protein structure determination, because they result in a loss of signals due to rapidly exchanging backbone amides and poor signal-to-noise of resonances. Therefore, a non-uniform sampling (NUS) scheme had to be employed to provide sufficient resolution (Mobli, 2015 ▸). More recently, the same group also reported the NMR structure of the wild-type MAL TIR domain at physiological pH (7.5) (Rahaman *et al.*, 2024 ▸). MAL oligomerization was minimized by reducing protein concentration (∼106 µ*M* versus 300 µ*M* for the C116A mutant) and by using a very low salt concentration (10 m*M* NaCl).

In contrast to the crystal structures, all the cysteine residues are reduced in the two NMR structures of MAL, resulting in a more characteristic TIR-domain fold (Fig. 2 ▸), suggesting that the unusual conformation observed in the crystal structures was a consequence of di­sulfide bond formation during the crystallization process. The BB loop and αB helix are highly disordered in the structure of the MAL C116A mutant, but adopt a more ordered conformation in the wild-type MAL NMR structure.

NMR redox measurements revealed that C91 has the highest redox potential of all cysteines in MAL. The resonance assignments for MAL TIR domain also enabled the use of NMR to characterize the binding site of the small-molecule inhibitor o-vanillin, which showed that this compound forms a covalent bond with Lys210 of MAL (Rahaman *et al.*, 2024 ▸).

The NMR structure of the TLR1 TIR domain is similar to the reported crystal structures, but as observed in NMR structures of TLR adaptors, several of the loop regions, including the BB and CD loops, adopt a more disordered structure (Lushpa *et al.*, 2021 ▸) [Fig. 2 ▸(*e*)].

## 4. Enzymatic activity of TIR domains and the use of NMR spectroscopy to identify nucleotide products

**Figure 3:**
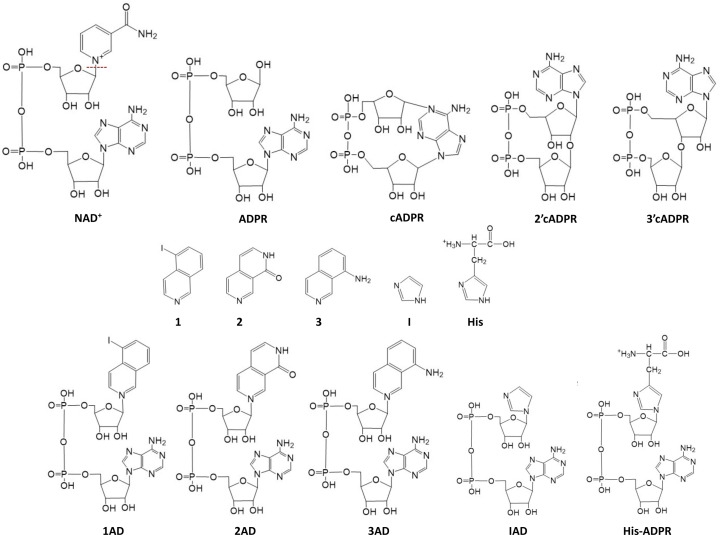
Chemical structures of nucleotides produced by TIR domain enzymes. ADPR, cADPR, 2′cADPR and 3′cADPR are produced by TIR domain-catalyzed hydrolysis of NAD^+^. Several TIR domains can also catalyze base-exchange reactions where the nicotinamide moiety of NAD^+^ is replaced by various heterocyclic amines such as **1** (5-iodo-iso­quinoline), **2** (1,2-di­hydro-2,7-naphthyridin-1-one), **3** (8-amino-iso­quinoline), imidazole (I) and histidine, yielding **1AD**, **2AD**, **3AD**, **IAD** and His-ADPR, respectively. The red line in NAD^+^ shows the cleavage site that removes the nicotinamide moiety from the ADPR.

^1^H NMR is a useful technique to study enzyme kinetics because the change in concentration of both substrates and products can be monitored in real time and multiple reactions can be followed simultaneously (Vang *et al.*, 2022 ▸). ^1^H NMR has been used for characterizing the multiple reactions that TIR domains catalyze: hydrolysis of NAD^+^ into nicotinamide and adenosine diphosphate ribose (ADPR) (Horsefield *et al.*, 2019 ▸; Shi *et al.*, 2022 ▸), cyclization of NAD^+^ into cyclic ADPR (cADPR) (Manik *et al.*, 2022 ▸), and base exchange of the nicotinamide moiety of NAD^+^ with various heterocyclic amines and prodrugs (Shi *et al.*, 2022 ▸) (Fig. 3 ▸).

NMR has also been used to determine the chemical structures of two cyclic ADPR (cADPR) isomers (2′cADPR and 3′cADPR) that are produced by some bacterial and plant TIR domains (Manik *et al.*, 2022 ▸) (Fig. 3 ▸). 3′cADPR is a recently discovered immune signalling molecule that activates anti-phage defence in bacteria and suppresses immunity in plants (Manik *et al.*, 2022 ▸; Ofir *et al.*, 2021 ▸; Leavitt *et al.*, 2022 ▸). To determine the chemical structures of these molecules, 2′cADPR and 3′cADPR were produced by the bacterial TIR domain-containing proteins AbTir (from *Acinetobacter baumannii*) and AaTir (from *Aquimarina amphilecti*), purified from the reaction mixtures using HPLC (high-performance liquid chromatography), and characterized by heteronuclear multiple bond correlation (HMBC) NMR. The HMBC spectra showed that 2′cADPR has cross-peaks (between H1′′–C2′ and H2′–C1′′), consistent with the formation of a ribose(1′′→2′)ribose *O*-glycosidic linkage, while 3′cADPR has cross-peaks (between H1′′–C3′ and H3′–C1′′) indicating the formation of a ribose(1′′→3′)ribose *O*-glycosidic linkage (Fig. 3 ▸). The two isomers were named 2′cADPR and 3′cADPR to highlight the linkages between the ribose rings in these molecules.

## 5. Reconstitution of filamentous complexes and structural characterization by cryo-EM

**Figure 4:**
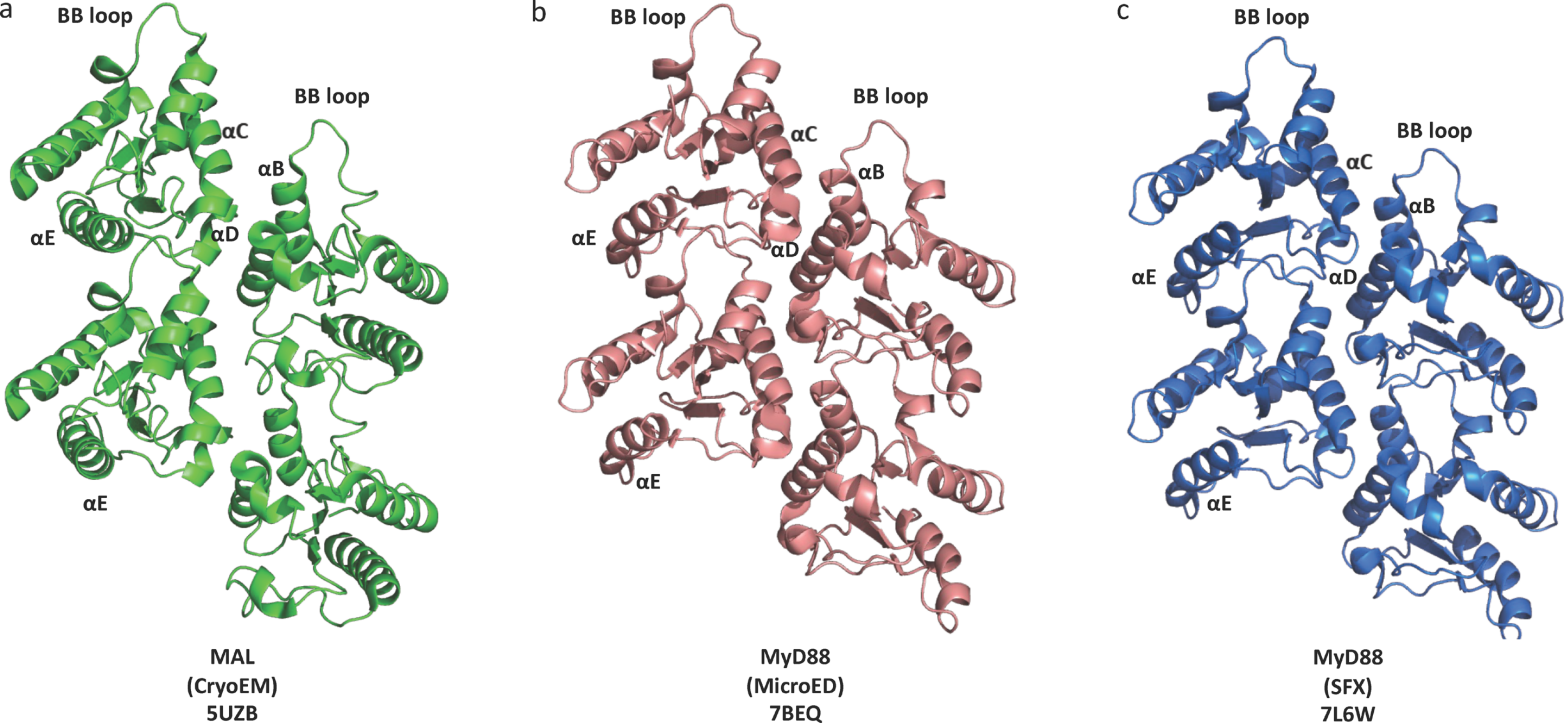
Higher-order assemblies of TLR adaptor TIR domains. (*a*) MAL (green), and (*b*) MyD88 (pink), microED structure; (*c*) MyD88 (blue), SFX structure TIR domains form similar parallel two-stranded assemblies.

NMR experiments on the MAL adaptor protein were made difficult by the tendency of the protein to precipitate at temperatures required for data collection, but a surprising finding was that the precipitated protein returns to solution at lower temperatures (Ve *et al.*, 2017 ▸; Hughes *et al.*, 2017 ▸). This observation led to a key discovery about TIR domains – that they form higher-order assemblies. It turns out that the precipitated protein formed filamentous assemblies, which were visible using negative-stain electron microscopy. A moderate-resolution structure (∼7 Å) could be determined with cryo-EM and helical reconstruction (Ve *et al.*, 2017 ▸). Filamentous helical assemblies were also observed for the mixture of TIR domains of TLR4 and MAL (Ve *et al.*, 2017 ▸). Structure-guided mutagenesis followed by cell signalling assays suggested that the biologically relevant part of the filament corresponds to two strands of TIR domains arranged in a head-to-tail manner, with the strands arranged parallel to each other and offset by half a TIR domain in the direction of the filament (described herein as a ‘parallel two-stranded assembly’) [Fig. 4 ▸(*a*)].

In some bacterial CBASS systems, TIR domains are fused to cyclic nucleotide binding domains such as STING (stimulator of interferon genes) and SAVED (SMODS-associated and fused to various effector domains) domains, which form filaments upon cyclic nucleotide binding; this association brings together TIR domains and activates their NADase function (Hogrel *et al.*, 2022 ▸; Morehouse *et al.*, 2022 ▸). In the PYCSAR system, the TIR domain-containing PycTIR protein also oligomerizes upon cUMP binding, similarly activating the TIR domain NADase activity (Tal *et al.*, 2021 ▸). Cryo-EM structures of TIR-STING and TIR-SAVED filaments reveal a head-to-tail arrangement of TIR domains as observed in the MAL filament. However, the TIR-STING filament consists of two antiparallel strands of TIR domains, while the TIR-SAVED filament features a single strand of TIR domains stabilized by SAVED domains, arranged head-to-tail.

## 6. Filaments that arrange into microcrystals

### 6.1. Structural characterization of microcrystals by microED

The TIR domain of the TLR adaptor MyD88 also forms insoluble assemblies, when combined with small amounts of MAL (Ve *et al.*, 2017 ▸). However, negative-stain EM revealed that these assemblies correspond to microcrystals, rather than filaments. The crystals were too small for conventional X-ray crystallography, so the techniques of microED and serial crystallography with an X-ray free-electron laser (XFEL) were employed in parallel.

MicroED is an emerging method that enables structure determination of macromolecules from sub-micrometre sized crystals (Shi *et al.*, 2013 ▸; Nannenga & Gonen, 2019 ▸; Clabbers *et al.*, 2021 ▸; Xu *et al.*, 2019 ▸; Shiriaeva *et al.*, 2023 ▸). The MAL-induced MyD88 microcrystals were of ideal size for this approach (Clabbers *et al.*, 2021 ▸) [Fig. S1(*a*) of the supporting information]. These microcrystals, however, have a tendency to aggregate, forming large bundles; a unique advantage of microED over techniques such as XFEL and micro-focused X-ray synchrotron beamlines is that single crystals can be identified to collect diffraction data (Sasaki *et al.*, 2023 ▸; Clabbers *et al.*, 2021 ▸; Roslova *et al.*, 2020 ▸). Typically, MyD88^TIR^ microcrystals diffract to around 2.8 Å resolution [Fig. S1(*c*)].

### 6.2. Structural characterization of microcrystals by serial crystallography

In parallel, the structure of MAL-induced MyD88 TIR domain microcrystals was solved using serial femtosecond crystallography (SFX) (Clabbers *et al.*, 2021 ▸). SFX exploits the femtosecond-scale duration of extremely brilliant XFEL pulses for the collection of diffraction data at room temperature (Boutet *et al.*, 2012 ▸; Chapman *et al.*, 2011 ▸; Schlichting, 2015 ▸; Spence, 2017 ▸). Diffraction data are collected as single snapshots from randomly oriented microcrystals, before they are destroyed by the radiation (Chapman *et al.*, 2011 ▸; Spence, 2017 ▸). Initially, serial crystallography was attempted on a fixed target at the synchrotron PETRAIII PII beamline; however, bundling of microcrystals into larger aggregates prevented collection of diffraction data of sufficient quality. Successful data collection was accomplished with the sample delivered as a stream of solvated microcrystals with a gas-dynamic virtual nozzle injector (DePonte *et al.*, 2008 ▸; Weierstall *et al.*, 2012 ▸) to a pulsed XFEL beam at the Linac Coherent Light Source (LCLS), SLAC National Acceleratory Laboratory (Liang *et al.*, 2015 ▸).

Both the microED and the SFX approaches produced virtually identical crystal structures at 3 and 2.3 Å resolution, respectively [Figs. 4 ▸(*b*)–4 ▸(*c*)]. The structures featured filamentous assemblies of MyD88 TIR domains analogous to the parallel two-stranded assembly of MAL TIR domains; these filamentous assemblies stack into crystalline arrays. Analogous assemblies are also present in the crystals of TRR-2 [PDB entry 4w8g; Fig. 1 ▸(*e*)] that were studied by conventional X-ray crystallography, as mentioned above. Structure-guided mutagenesis followed by cell signalling assays again confirmed that that the BE and BCD interfaces observed in the two-stranded assemblies of MyD88 TIR domains correspond to the interfaces formed during signalling in the cell.

## 7. Structural characterization of full-length proteins by single-particle cryo-EM

**Figure 5:**
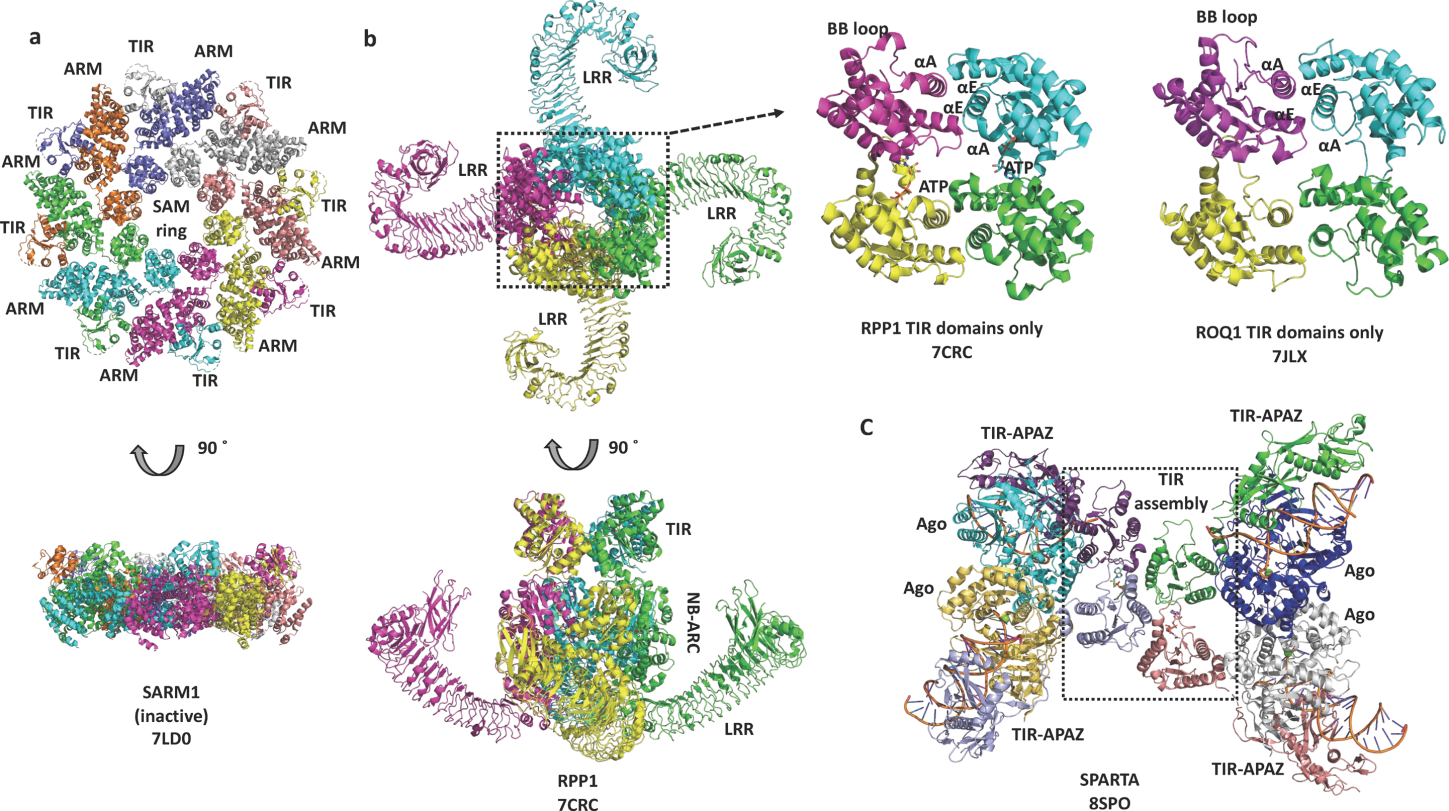
Cryo-EM structures of SARM1 and plant TNLs. SARM1 forms a double-layered, octameric ring. The TNLs RPP1 and ROQ1 form tetramers with their TIR domains in an anti-parallel two-stranded assembly. ATP is shown in stick representation. Chains are shown in different colours.

The technical improvements in cryo-EM in the last decade facilitated the study of 3D structures of full-length TIR domain-containing proteins at high resolution (Table S1). In a short period of time, SARM1 became one of the best structurally characterized TIR domain-containing proteins, largely because of the interest in this protein as a target for neurodegenerative disease (Coleman & Höke, 2020 ▸; DiAntonio *et al.*, 2021 ▸; McGuinness *et al.*, 2023 ▸). SARM1 contains three key functional regions: the N-terminal armadillo-repeat motif (ARM) domain, the central tandem sterile alpha motif (SAM) domains and a C-terminal TIR domain. Several groups determined the structure of the protein in its inactive state, revealing an octameric ring formed by the tandem SAM domains; the next layer in the ring formed by the ARM domains extending out from the central ring; and the TIR domains wedged between the ARM domains on the outside of the ring (Figley *et al.*, 2021 ▸; Jiang *et al.*, 2020 ▸; Shen *et al.*, 2021 ▸; Bratkowski *et al.*, 2020 ▸; Sporny *et al.*, 2020 ▸; Li *et al.*, 2021 ▸) [Fig. 5 ▸(*a*)]. Because the TIR domains are kept apart in this arrangement, they cannot exert their NAD^+^ hydro­lase activity. Initial attempts to observe the active state of the protein upon addition of the activating molecule NMN (nicotinamide mono-nucleotide) resulted in the ‘disappearance’ of the TIR domains from the ring, and only the SAM and ARM domains could be visualized in the cryo-EM images. The stabilization of the TIR-domain structure in the active state required the use of chemical ‘glue’ described below.

Cryo-EM further facilitated structure determination of the activated TIR domain-containing plant NLRs (TNLs) ROQ1 from *Nicotiana benthamiana* and RPP1 from Arabidopsis (Martin *et al.*, 2020 ▸; Ma *et al.*, 2020 ▸). TNLs contain an N-terminal TIR domain, a central NB-ARC (nucleotide-binding domain shared with APAF1, R gene products and CED4) domain and a C-terminal LRR (leucine-rich repeat) domain. When binding to their activating ligand protein (‘effector’) from a pathogen, they assemble into a tetrameric assembly called the ‘resistosome’. Despite the fourfold rotational symmetry of the ring formed by the NB-ARC and LRR domains, the TIR domains sit on top of this ring and form their own assembly with a different symmetry, corresponding to a mini-filament featuring two TIR domains in a head-to-tail arrangement, and the other two TIR domains associating in an antiparallel manner, forming an ‘antiparallel two-stranded’ assembly [Fig. 5 ▸(*b*)].

The SPARTA system detects invading plasmids in bacteria. The TIR-APAZ and short prokaryotic Argonaute (pAgo) proteins form a heterodimeric complex that cleaves NAD^+^ upon DNA recognition via a guide RNA (Koopal *et al.*, 2022 ▸). Several recent cryo-EM studies have demonstrated that binding of a guide RNA:target ssDNA duplex leads to the formation of a TIR-APAZ:pAgo tetramer consisting of two parallel head-to-tail-organized TIR dimers, analogous to the arrangement of TIR domains observed in the MAL and AbTir filaments, described below (Guo *et al.*, 2023 ▸, 2024 ▸; Gao *et al.*, 2024 ▸; Kottur *et al.*, 2023 ▸; Ni *et al.*, 2023 ▸; Finocchio *et al.*, 2024 ▸; Shen *et al.*, 2023 ▸; Wang *et al.*, 2023 ▸) [Fig. 5 ▸(*c*)]. The head-to-tail association of TIR domains leads to the formation of the substrate NAD^+^-binding pockets (see below) and the activation of SPARTA’s NADase activity. Similar to SARM1 and plant NLRs, the SPARTA immune system is kept inactive by preventing their TIR domains from self-associating.

## 8. Use of NAD^+^ mimetics to stabilize the active conformation of TIR-domain enzymes

**Figure 6:**
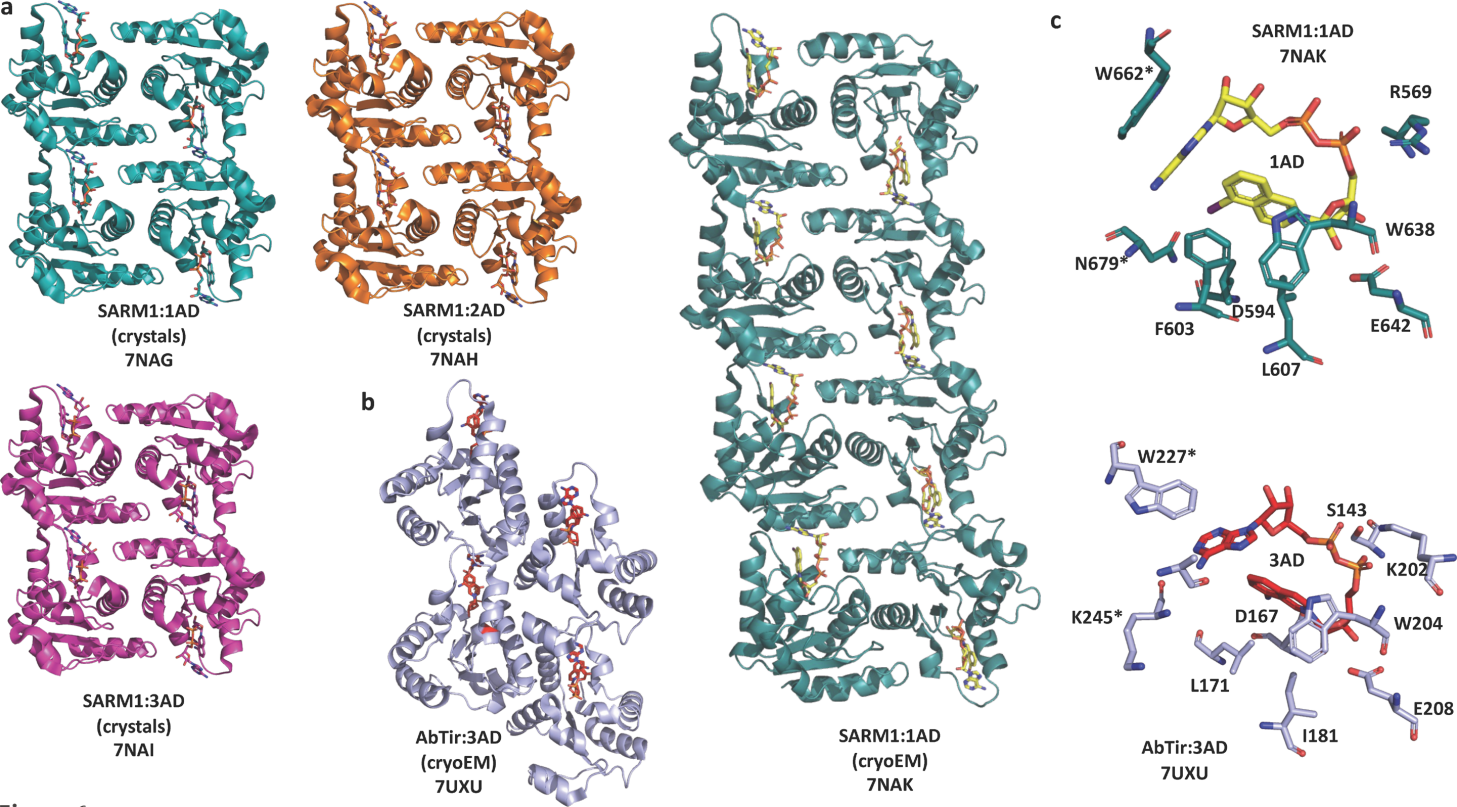
Structures of TIR domain enzyme assemblies stabilized by chemical ‘glues’. (*a*) Crystal structures of SARM1 TIR domain in complex with **1AD** (teal), **2AD** (orange) and **3AD** (magenta), respectively. (*b*) Cryo-EM structures of the TIR domain assembly of AbTir (light-purple cartoon) and SARM1 (teal cartoon) stabilized by **3AD** (red stick) and **1AD** (yellow stick), respectively. (*c*) Stick representation of the active sites in SARM1 and AbTir. Residues with * are from the adjacent TIR domains.

The base-exchange activity observed for the SARM1 TIR domain has been essential to understand the mechanism of action of some early inhibitors of axon degeneration (Hughes *et al.*, 2021 ▸) and to advance the structural studies of active assemblies of TIR domains. When supplied an appropriate ‘base’ [*e.g.* compounds **1** (5-iodo-iso­quinoline), **2** (1,2-di­hydro-2,7-naphthyridin-1-one) or **3** (8-amino-iso­quinoline)], the base-exchange reaction substitutes the nicotinamide group with the base to produce dinucleotides such as **1AD** (5-iodo-iso­quinoline adenine dinucleotide [AD]), **2AD** (1,2-di­hydro-2,7-naphthyridin-1-one AD) and **3AD** (8-amino-iso­quinoline AD), respectively (Shi *et al.*, 2022 ▸) (Fig. 3 ▸). These NAD^+^ mimetics are more biochemically stable than NAD^+^ itself and have found use as molecular ‘glue’ to stabilize the active assemblies of TIR domains. For example, each of the three compounds was captured in crystals of SARM1 TIR domain when soaked with NAD^+^ and compounds **1**–**3** [Fig. 6 ▸(*a*)]. The structures revealed that the active site of SARM1 spans two TIR domain molecules, explaining the requirement of TIR-domain self-association for NADase activity. Given the similarity of these mimetics to NAD^+^, it is likely that NAD^+^ would have a similar binding mode. Furthermore, **1AD**, together with the activator NMN, was utilized to determine cryo-EM structures of human SARM1 in the active state (Shi *et al.*, 2022 ▸), while **3AD** was used to capture the bacterial protein AbTir in the active filamentous state and the structure was determined by helical reconstruction cryo-EM (Manik *et al.*, 2022 ▸) [Fig. 6 ▸(*b*)]. In SARM1, the assembly architecture features the antiparallel two-stranded assembly analogous to the one observed for the TIR domains in TNL resistosomes and TIR-STING filaments. The binding mode of **3AD** in the AbTir TIR domain filament structure is also very similar, although TIR domains in this case assemble into a parallel two-stranded assembly analogous to the TIR domains from the TLR adaptor proteins MAL and MyD88, and the SPARTA immune system [Fig. 6 ▸(*c*)]. The catalytic reaction is likely to occur via a covalent glycosyl–enzyme complex intermediate [based on studies on SARM1 (Shi *et al.*, 2022 ▸)]. The binding mode with the two bases of the dinucleotide placed close to each other is consistent with the ability of TIR domains to yield cyclic ADPR products. The TIR domains from the type II Thoeris antiphage defence system have recently been shown to exchange nicotinamide for imidazole or histidine, producing imidazole adenine dinucleotide (IAD) and His-ADPR, respectively (Shi *et al.*, 2024 ▸; Sabonis *et al.*, 2024 ▸) (Fig. 3 ▸).

## 9. General implications

Common features of assemblies observed for various TIR domains only begin to emerge when we compare the different structures. The first key observation is that all active signalling assemblies feature an open-ended head-to-tail strand of TIR domains. The interface responsible for this association is the BE interface that includes the well characterized BB loop. The active site in the TIR domains featuring enzymatic activity is found between two TIR domains in the BE interface. Enzymatic activity therefore depends on the association through this interface (Fig. 6 ▸).

The head-to-tail association of TIR domains in a single strand presumably features low affinity and is transient. In agreement, these single strands are always stabilized in some way through additional interfaces. This can be accomplished by two such strands of TIR domains associating in a parallel fashion (two-stranded parallel assembly, as observed for the TLR adaptors MAL and MyD88, as well as the bacterial protein AbTir) or antiparallel fashion (two-stranded antiparallel assembly, as observed for SARM1, plant TIR domains and TIR-STING). This can also be accomplished with the help of other domains, as observed in the bacterial TIR-SAVED proteins.

Despite the individual TIR-domain strands being stabilized through higher-order association in the active signalling assemblies, individual TIR domains in solution show limited self-association tendency and largely behave as monomeric proteins. In the cell though, the domains are found as part of larger multi-domain proteins and function within the context of multi-step signalling pathways; it is only when considering this cellular context that the molecular behaviour of TIR domains starts to make sense. The activation of immune and cell-death pathways is only beneficial to cells in very specific circumstances, and therefore the TIR domains must be kept in an inactive state until the cell faces an emergency. It is this additional molecular and cellular context that regulates the activity of TIR domains and only allows them to be activated when necessary.

**Figure 7:**
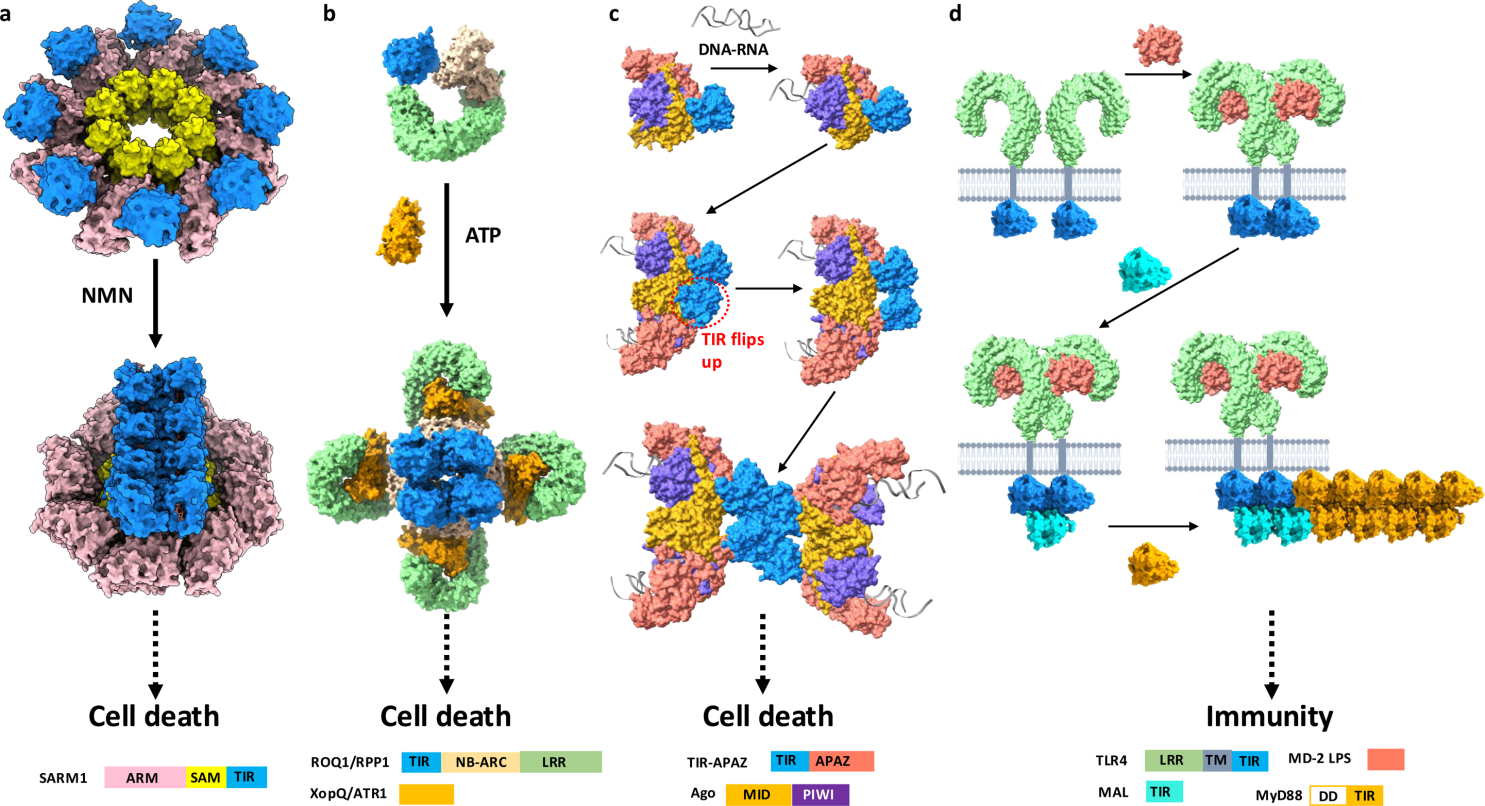
Mechanisms of regulation of TIR-domain assembly in immunity and cell-death pathways: (*a*) SARM1, (*b*) plant TNLs, (*c*) the SPARTA anti-phage defence pathway and (*d*) SCAF in TLR pathways [illustrated by TLR4 signalling through the adaptors MAL and MyD88; MyD88 contains a death domain and the assembly of TIR domains is proposed to recruit protein kinases from the IRAK family the ubiquitin ligase TRAF6 (tumour necrosis factor receptor-associated factor 6) and activate them through proximity-based activation (not shown); the recruitment of the adaptor MAL by dimerized TLR4 TIR domains, and the recruitment of the adaptor MyD88 by the TLR4:MAL assembly represent nucleation-controlled steps in assembly formation].

There are two key mechanisms that control this assembly (Fig. 7 ▸). The first is the presence of other domains in the TIR domain-containing proteins; these additional domains oligomerize and bring the TIR domains closer together, increasing their effective concentration [Figs. 7 ▸(*a*)–7 ▸(*c*)]. In the case of SARM1 [Figs. 5 ▸(*a*) and 7 ▸(*a*)], for example, the SAM domains always exist as an octameric ring. In the inactive state, the TIR domains are prevented from self-association by the ARM domains keeping them apart. The binding of the activating small-molecule NMN to the ARM domains causes the TIR domains to dissociate from the ARM domains, and they become free to self-associate into an active enzymatic two-stranded antiparallel assembly. By contrast, TNLs [Figs. 5 ▸(*b*) and 7 ▸(*b*)] exist as monomers, and assemble into tetramers only upon effector binding, through the association of NB-ARC and LRR domains. This now brings the TIR domains together so they can form an active enzymatic assembly equivalent to the SARM1 TIR domains. In the bacterial CBASS system, nucleotide binding to SAVED and STING domains induces filament formation. There is also a report of TIR-domain assembly facilitated by double-stranded DNA, although the biological implications of this structure remain controversial (Yu *et al.*, 2022 ▸; Tian *et al.*, 2022 ▸).

The second mechanism of regulation is the nucleated hierarchical mechanism we call SCAF [signalling by cooperative assembly formation, see Fig. 7 ▸(*d*)] (Nimma *et al.*, 2017 ▸). Physical principles dictate that, at a sufficiently high concentration, open-ended higher-order assemblies like filaments will self-assemble. To do this, they must overcome an energy barrier imposed by entropy and fulfil other parameters specific to a particular assembly, such as conformational changes and cooperativity. In the cell, we envision TIR domain-containing proteins such as TLR adaptors exist at a concentration associated with a metastable state, poised to associate, but unable to overcome the energy barrier. However, once provided a seed (*e.g.* dimerized TLR), the TLR adaptor can overcome this energy barrier and start associating into its open-ended assembly and thereby start the signalling process (Rodriguez Gama *et al.*, 2021 ▸) [Fig. 7 ▸(*d*)]. The growth of the assembly will stop when the adaptor protein is depleted or physical restrictions prevent further growth.

There is one crystal structure of a heterodimer of TIR domains that we have not discussed so far. This is the only available structure of a heteromeric complex of TIR domains, and corresponds to the heterodimer of TIR domains from the Arabidopsis TNLs RPS4 and RRS1 (Williams *et al.*, 2014 ▸). These two proteins function as a pair, with only RPS4 thought to be the signalling receptor. Although the mechanism of regulation of paired receptors is not well understood (Yang *et al.*, 2024 ▸), based on what we know about the better-characterized TNLs (Martin *et al.*, 2020 ▸; Ma *et al.*, 2020 ▸), we postulate that the TIR domain hetero-complex represents an inactive state, where the association needs to be broken and the RPS4 TIR domains allowed to self-associate to start signalling. Alternatively, the TIR-domain hetero-complex could self-associate directly into the active structure.

The studies on TIR domains effectively highlight the complementarity of structural approaches. Each technique clearly has advantages and limitations, and only the combination and integration of different approaches allowed us to build a comprehensive understanding of the molecular mechanisms associated with functions of TIR domains across phyla. For example, crystal contact analyses failed to identify all biologically meaningful protein:protein interfaces and were often misleading. Similarly, di­sulfide bonds are unusual in cytoplasmic proteins and those found in TIR domain structures may be the result of oxidizing conditions during prolonged crystallization experiments. The work also highlights the importance of functional validation of structural data; the nature and size of TIR-domain assemblies reconstituted *in vitro* do not always reflect the signalling assemblies found in cells, but structure based mutagenesis coupled with functional assays can uncover which interfaces correspond to the ones that occur in cells.

Past computational approaches that attempted to predict the signalling complexes of TIR domains based on early crystal structures [reviewed by Nimma *et al.* (2017 ▸)] largely failed, as demonstrated by subsequent structures of reconstituted assemblies characterized mostly by cryo-EM (Ve *et al.*, 2017 ▸). It is tempting to revisit now if active signalling assemblies can be predicted based on the new structural knowledge and the availability of new structure predictions methods such as *AlphaFold*2 (Jumper *et al.*, 2021 ▸) and *AlphaFold*3 (Abramson *et al.*, 2024 ▸); the latter can take into account ligands such as NAD^+^.

## 10. Related literature

The following references are cited in the supporting information: Bratkowski *et al.* (2022 ▸); Burdett *et al.* (2021 ▸); Chan *et al.* (2010 ▸); Halabi *et al.* (2017 ▸); Hattne *et al.* (2018 ▸); Hou *et al.* (2022 ▸); Hyun *et al.* (2016 ▸); Jang & Park (2014 ▸); Khan *et al.* (2004 ▸); Khazma *et al.* (2022 ▸; 2023 ▸); Klontz *et al.* (2023 ▸); Ko *et al.* (2023 ▸); Kottur *et al.* (2024 ▸); Nannenga *et al.* (2014 ▸); Nimma *et al.* (2022 ▸); Nyman *et al.* (2008 ▸); Snyder *et al.* (2013 ▸); Tao *et al.* (2002 ▸); Zhang *et al.* (2024 ▸); Zhou *et al.* (2022 ▸).

## Supplementary Material

Supporting table and figure. DOI: 10.1107/S2052252524007693/ur5004sup1.pdf

## Data Availability

The authors confirm that the data supporting the findings of this study are available within the article and its supplementary materials.
