# Supplementary material for: Structural characterization of TIR-domain signalosomes through a combination of structural biology approaches
Source: IUCrJ. 2024 Aug 27;11(Pt 5):695–707. doi: 10.1107/S2052252524007693 (PMC11364022; doi:10.1107/S2052252524007693)
Supplement: Supplementary file 1 [file m-11-00695-sup1.pdf]

IUCrJ

Volume 11 (2024)

Supporting information for article:

Structural characterization of TIR-domain signalosomes through a combination of structural biology approaches

Akansha Bhatt, Biswa P. Mishra, Weixi Gu, Mitchell Sorbello, Hongyi Xu, Thomas Ve and Bostjan Kobe

Table S1 TIR-domain structures

Protein name	Organism	PDB code	References
**X-ray crystallography**			
TLR1 TIR domain	*Homo sapiens*	1FYV<br>7NUW (crystallized with Zn^{2+} ions)<br>7NUX	(Xu *et al.*, 2000, Lushpa *et al.*, 2021)
TLR2 TIR domain	*Homo sapiens*	1FYW,	(Tao *et al.*, 2002, Xu *et al.*, 2000)
		1FYX (P681H mutant)	
		1077 (C713S mutant)	
TLR6 TIR domain	*Homo sapiens*	4OM7	(Jang & Park, 2014)
TLR10 TIR domain	*Homo sapiens*	2J67	(Nyman *et al.*, 2008)
TLR15 TIR domain	*Gallus gallus*	7YLG (glutathione adduct)	(Ko *et al.*, 2023)
		7YLF (2-mercaptoethanol adduct)	
IL-1RAPL1 (IL-1R9) TIR domain	*Homo sapiens*	1T3G	(Khan *et al.*, 2004)
IL-1RAPL2 (IL-1R10) TIR domain	*Homo sapiens*	7FD3<br>7SZL	(Nimma *et al.*, 2022, Zhou *et al.*, 2022)
IL-18Rβ (IL-1R7) TIR domain	*Homo sapiens*	7FCH	(Zhou *et al.*, 2022)
IL-1RAcPb (IL-1R3b) TIR domain	*Homo sapiens*	7FCC	(Zhou *et al.*, 2022)
SIGIRR (Il-1R8) TIR domain	*Danio rerio*	7FCL	(Zhou *et al.*, 2022)
		7FCJ (C299S mutant)	
SARM1 TIR domain	*Homo sapiens*	6O0V, 6O1B (G601P mutant)<br>6O0U (H685A mutant)<br>6O0Q (ribose)<br>6O0R (glycerol)<br>7NAG (1AD)<br>7NAH (2AD)	(Bratkowski *et al.*, 2022, Horsefield *et al.*, 2019, Shi *et al.*, 2022)

		7NAI (3AD)<br>7NAJ (ara-2'F-ADPR)<br>8D0J (ligand-free)<br>8D0C (NB-3-ADPR)<br>8D0D (NB-7-ADPR)<br>8D0E (NB-7)<br>8D0F (NB-2-ADPR)<br>8D0G (NB-3-ADPRP)<br>8D0H (NB-3-GDPR)<br>8D0I (NB-3-eADPR)	
MyD88 TIR domain	*Homo sapiens*	4DOM<br>4E07	(Snyder *et al.*, 2013)
MAL TIR domain	*Homo sapiens*	2Y92<br>3UB2<br>3UB3 (D96N mutant)<br>3UB4 (S180L mutant)<br>4FZ5<br>4LQD	(Lin *et al.*, 2012, Snyder *et al.*, 2014, Valkov *et al.*, 2011, Woo *et al.*, 2012)
BCAP TIR domain	*Homo sapiens*	5FOR	(Halabi *et al.*, 2017)
TRR-2 TIR domain	*Hydra magnipapillata*	4W8G, 4W8H	
TIR-STING	*Crassostrea gigas*	6WT6<br>6WT7 (2',3'-cGAMP)	(Morehouse *et al.*, 2020)
AtTIR	*Arabidopsis thaliana*	3JRN	(Chan *et al.*, 2010)
L6 TIR domain	*Linum usitatissimum*	3OZI	(Bernoux *et al.*, 2011)
RUN1 TIR domain	*Vitis rotundafolia*	6O0W ($NADP^+$ and Bis-Tris)<br>7RX1<br>7RTS<br>7S2Z (E100A mutant)	(Horsefield *et al.*, 2019, Burdett *et al.*, 2021)
RRS1:RPS4 TIR domain complex	*Arabidopsis thaliana*	4C6T	(Williams *et al.*, 2014)

RPS4 TIR domain	*Arabidopsis thaliana*	4C6R	(Williams *et al.*, 2014)
RRS1 TIR domain	*Arabidopsis thaliana*	4C6S	(Williams *et al.*, 2014)
RPV1 TIR domain	*Muscadinia rotundafolia*	5KU7	(Williams *et al.*, 2016)
SNC1 TIR domain	*Arabidopsis thaliana*	5TEC, 5H3C	(Hyun *et al.*, 2016, Zhang *et al.*, 2017)
RPP1 TIR domain	*Arabidopsis thaliana*	5TEB<br>7XOZ (ADPR)	(Jia *et al.*, 2022, Zhang *et al.*, 2017)
Eubrec_0645	*Eubacterium rectale*	3HYN	
PdTLP TIR domain	*Paracoccus denitrificans*	3H16	(Chan *et al.*, 2009)
Brucella effector BtpA/Btp1/TcpB TIR domain	*Brucella melitensis*	4LQC, 4LZP, 4C7M	(Alaidarous *et al.*, 2014, Kaplan-Turkoz *et al.*, 2013, Snyder *et al.*, 2014)
BtTir TIR domain	*Bacteroides thetaiotaomicron*	7UXR	(Manik *et al.*, 2022)
AbTir TIR domain	*Acinetobacter baumannii*	7UWG, 8G83	(Klontz *et al.*, 2023, Manik *et al.*, 2022)
ThsB TIR domain	*Bacillus cereus MSX-D12*	6LHY	(Ka *et al.*, 2020)
EcThsB2	*Escherichia coli*	8V6T	
SPARTA complex	*Thermoflavifilum*	8U7B	(Kottur *et al.*, 2024)

	thermophilum		
**SFX**			
MyD88 TIR domain	*Homo sapiens*	7BER, 7L6W	(Clabbers *et al.*, 2021)
**NMR**			
MyD88 TIR domain	*Homo sapiens*	2JS7, 2Z5V	(Ohnishi *et al.*, 2009)
MAL TIR domain	*Homo sapiens*	8JZM<br>2NDH (C116A mutant)	(Hughes *et al.*, 2017, Rahaman *et al.*, 2024)
TRAM TIR domain	*Homo sapiens*	2M1W	(Enokizono *et al.*, 2013)
TRIF TIR domain	*Homo sapiens*	2M1X	(Enokizono *et al.*, 2013)
TLR1 TIR domain	*Homo sapiens*	7NT7	(Lushpa *et al.*, 2021)
**MicroED**			
MyD88 TIR domain	*Homo sapiens*	7BEQ	(Clabbers *et al.*, 2021)
**CryoEM**			
MAL TIR domain	*Homo sapiens*	5UZB	(Ve *et al.*, 2017)
SARM1 (inactive state)	*Homo sapiens*	6WPK<br>6ZFX<br>7CM5<br>7LD0<br>7KNQ<br>7QG0 (TK106-induced duplex)<br>7DJT (dHNN adduct)<br>7ANW (NAD^+)<br>7CM6 (NAD^+)<br>7CM7 (E642A mutant, NAD^+)	(Bratkowski *et al.*, 2020, Figley *et al.*, 2021, Jiang *et al.*, 2020, Khazma *et al.*, 2022, Li *et al.*, 2021, Shen *et al.*, 2021, Sporny *et al.*, 2020)
SARM1 (active state)	*Homo sapiens*	7NAK (1AD)<br>8GNI (NMN and Nanobody-C6)<br>8GNJ (NMN and Nanobody-C6)	(Hou *et al.*, 2022, Shi *et al.*, 2022)
CeTIR-1	*C. elegans*	8P2M	(Khazma *et al.*, 2023)

		8P2L	
L7 TIR domain (dsDNA complex)	*Linum usitatissimum*	7VU8<br>7X5K<br>7X5L<br>7X5M	(Yu *et al.*, 2022)
RPP1	*Arabidopsis thaliana,*	7DFV<br>7CRC	(Ma *et al.*, 2020)
ROQ1	*Nicotiana benthamiana*	7JLX	(Martin *et al.*, 2020)
TIR-STING	*Sphingobacterium faecium*	7UN8 (c-di-GMP, single fiber)	(Morehouse *et al.*, 2022)
TIR-SAVED	*Microbacterium terrae*	7QQK (cA3)	(Hogrel *et al.*, 2022)
AbTir TIR domain	*Acinetobacter baumannii*	7UXU (3AD)	(Manik *et al.*, 2022)
SPARTA complex	*Maribacter polysiphoniae*	8OZ6 (tetramer ligand free),<br>8OZI (tetramer pre-NAD$^+$ cleavage)<br>8OZF (tetramer, post-NAD$^+$ cleavage)<br>8OZG (tetramer, post-NAD$^+$ cleavage)<br>8OZC (monomer)<br>8OZD (dimer)<br>8FEX (monomer)<br>8FFI (tetramer)<br>8SP0 (dimer)<br>8SP3 (dimer)<br>8SPO (tetramer; NAD$^+$)<br>8SQU (monomer)<br>8I87 (tetramer)<br>8I88 (monomer)	(Ni *et al.*, 2023, Shen *et al.*, 2023, Wang *et al.*, 2023)

SPARTA complex	*Thermoflavifilum thermophilum*	8ISY (monomer)<br>8ISZ (monomer)<br>8IT0 (dimer)<br>8K9G (dimer)<br>8IT1 (tetramer)<br>8J84 (monomer)<br>8J9G (monomer)<br>8J9P (dimer)<br>8JAY (tetramer)<br>8IFK (monomer)<br>8K34 (monomer)<br>8IFL (tetramer)<br>8IFM (tetramer)<br>8U72 (tetramer)	(Gao *et al.*, 2024, Guo *et al.*, 2024, Shen *et al.*, 2023, Zhang *et al.*, 2024)
SPARTA complex	*Bacillales bacterium*	8QLO (monomer)<br>8QLP (tetramer)	(Finocchio *et al.*, 2024)

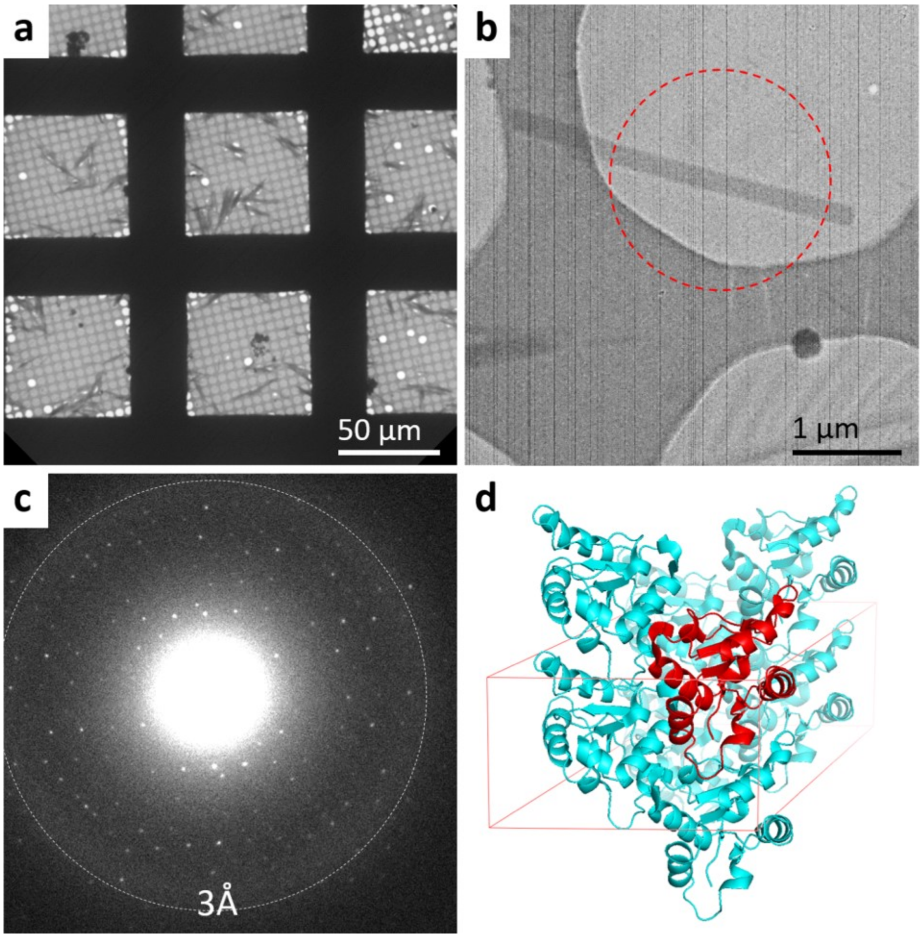

Figure S1 MicroED data collection of MyD88^{TIR} crystalline assemblies. (*a*) Overview of a suitable CryoEM grid of MyD88^{TIR} microcrystals. (*b*) A single crystal chosen for MicroED data collection. The red dotted circle illustrates the region isolated by the selective area aperture from where the MicroED data was collected. (*c*) A typical electron diffraction pattern in a continuous rotation MicroED dataset. (*d*) MyD88^{TIR} crystal structure highlighting an individual molecule and the unit cell. After a suitable crystal is found, MicroED data is collected by continuously rotating the crystal while recording the diffraction pattern simultaneously. This can be performed either in selective area diffraction mode or in nano-probe mode. MicroED data collection parameters should be carefully designed based on the properties of the crystal and availability of the instrument. In general, the accumulated electron dose should be kept below 5 $e^-/Å^2$, when working with protein crystals (Nannenga *et al.*, 2014, Hattne *et al.*, 2018). Therefore, when working with a mainstream transmission (cryo)-electron microscope equipped with scintillator-based CMOS (complementary metal-oxide semiconductor) detectors, it is recommended to collect several datasets with a tilt range of 40° to 60° per dataset. In order to improve the overall data completeness, these small wedges of data need to be collected over a large angular range, for example, tilting from -60° to -20°, -40° to 0°, -20° to 20°, 0° to 40°, and 20° to 60°. These datasets can be processed and merged using software developed for X-ray crystallography. Structure solution and refinement are then performed using X-ray crystallography software.
